# Prevalence of alcohol use disorder in migrants from a Portuguese Center for Refugees: a study protocol

**DOI:** 10.1192/j.eurpsy.2024.1273

**Published:** 2024-08-27

**Authors:** D. Magalhaes, P. Satuala, J. Bastos, S. Martins, M. Oliveira, T. Sousa, A. Neto

**Affiliations:** ^1^Mental Health Department, Hospital Prof. Doutor Fernando Fonseca; ^2^ Conselho Português para os Refugiados; ^3^Unidade de Alcoologia de Lisboa. Divisão de Intervenção nos Comportamentos Aditivos e nas Dependências. Administração Regional de Saúde de Lisboa e Vale do Tejo, Lisbon, Portugal

## Abstract

**Introduction:**

According to the 2023 statistical report from the Portuguese Migration Observatory, Portugal has received over 72,000 refugees since 2015, with a significant number (56,041) being displaced citizens from Ukraine. This influx includes spontaneous asylum requests. The major countries of origin for refugees in Portugal are Afghanistan, India, Gambia, Pakistan, and Morocco. Notably, refugee populations exhibit elevated incidence rates of specific psychiatric disorders, including post-traumatic stress disorder and depressive disorders. These conditions are independent risk factors for substance use disorders. Furthermore, refugees face unique risks related to their migration journey, increasing their vulnerability to substance use disorders. The prevalence of substance use disorders, especially Alcohol Use Disorder, can reach up to 36% in this population. CAR 1 (Reception Center for Refugees) is a vital social facility in Portugal dedicated to enhancing the reception and integration of asylum seekers and refugees.

**Objectives:**

Our primary objective is to determine the prevalence of potential alcohol problems and unhealthy alcohol use within the Portuguese Refugee Center in Lisbon. Our secondary aim is to comprehensively characterize the migrant population. This includes gathering data regarding demographic information, legal status, country of origin, pre-migration alcohol-related issues, psychiatric diagnoses, history of psychiatric evaluations, self-initiated help-seeking behavior, and self-perceived alcohol-related problems.

**Methods:**

All individuals currently residing in our refugee center (approximately 70 people) will be invited to participate in a comprehensive survey and screening process. Exclusion criteria will apply to individuals with acute psychiatric conditions unable to provide reliable responses. The survey includes the Alcohol Use Disorders Identification Test (AUDIT) and the CAGE questionnaire. Quantitative data obtained from the questionnaires will be analyzed using Microsoft Excel and IBM SPSS 29 software.

**Results:**

We anticipate a high prevalence of positive responses to the AUDIT due to potential alcohol-related issues but expect low responses to the CAGE questionnaire due to limited awareness of alcohol use disorder and a reduced perception of the need for help.

**Conclusions:**

This study could help identify and validate the prevalence of alcohol use disorders among migrants, emphasizing the need for appropriate responses. By shedding light on these challenges, we hope to promote effective responses to alcohol use disorder and encourage the utilization of alcohol screening tests in refugee centers, emphasizing the importance of seeking consultation when needed.

**Disclosure of Interest:**

None Declared

